# Supplementary material for: Calcium Signaling in the Cerebellar Radial Glia and Its Association with Morphological Changes during Zebrafish Development
Source: Int J Mol Sci. 2021 Dec 16;22(24):13509. doi: 10.3390/ijms222413509 (PMC8706707; doi:10.3390/ijms222413509)
Supplement: Supplementary file 1 [file ijms-22-13509-s001.zip › Supplementary information.pdf]

Supplementary information:

Table S1. Comparison of radial glia morphologies in the cerebellar regions at the different ages.

3dpf				
**Characteristics**	**Mann-Whitney test**	**Mean diff.**	**P Value**	**Summary**
Soma area	Central vs. lateral	-2.6	.049	*
Process Longitude	Central vs. lateral	-1.2	.790	ns
Number of processes	Central vs. lateral	0.0	.094	ns

5dpf					
**Characteristics**	**Tukey's multiple comparisons test**	**Mean Diff.**	**95.00% CI of diff.**	**Summary**	**Adjusted P Value**
Soma area	CCe vs. Va	1.444	-13.07 to 15.96	ns	0.994
	EG vs. Va	0.4621	-13.64 to 14.56	ns	>0.999
	LCa vs. Va	7.371	-7.147 to 21.89	ns	0.549
	EG vs. CCe	-0.9823	-15.50 to 13.54	ns	0.998
	LCa vs. CCe	5.927	-8.996 to 20.85	ns	0.728
	LCa vs. EG	6.909	-7.609 to 21.43	ns	0.601
Process Longitude	Va vs. CCa	-11.36	-27.15 to 4.425	ns	0.2422
	Va vs. EG	-5.476	-19.44 to 8.484	ns	0.7343
	Va vs. LCa	-15.46	-29.89 to -1.027	*	0.0309
	CCa vs. EG	5.888	-10.23 to 22.01	ns	0.7747
	CCa vs. LCa	-4.095	-20.63 to 12.44	ns	0.9159
	EG vs. LCa	-9.983	-24.78 to 4.813	ns	0.2965
Number of processes	CCa vs. Va	-0.4638	-0.9937 to 0.06610	ns	0.1065
	EG vs. Va	0.3308	-0.2193 to 0.8809	ns	0.3927
	LCa vs. Va	0.08882	-0.6187 to 0.4411	ns	0.9708
	EG vs. CCa	0.7946	0.2231 to 1.366	**	0.0028
	LCa vs. CCa	0.3750	-0.1772 to 0.9272	ns	0.2862
	LCa vs. EG	-0.4196	-0.9912 to 0.1519	ns	0.2227

7dpf				
**Characteristics**	**Dunn's multiple comparisons test**	**Mean rank diff.**	**Adjusted P Value**	**Summary**
Soma area	CCa vs. Va	-3.743	>0.9999	ns
	EG vs. Va	-4.676	>0.9999	ns
	LCa vs. Va	-6.520	>0.9999	ns
	EG vs. CCa	-0.9333	>0.9999	ns
	LCa vs. CCa	-2.777	>0.9999	ns
	LCa vs. EG	-1.844	>0.9999	ns
Processes Longitude	CCa vs. Va	29.58	0.1330	ns
	EG vs. Va	1.005	>0.9999	ns
	LCa vs. Va	9.071	>0.9999	ns
	EG vs. CCa	-28.58	0.1775	ns
	LCa vs. CCa	-20.51	0.7859	ns
	LCa vs. EG	8.066	>0.9999	ns
Number of processes	CCa vs. Va	-4.147	>0.9999	ns
	EG vs. Va	-0.2408	>0.9999	ns
	LCa vs. Va	-8.585	0.9772	ns
	EG vs. CCa	3.906	>0.9999	ns
	LCa vs. CCa	-4.438	>0.9999	ns
	LCa vs. EG	-8.344	>0.9999	ns

Table S2. Calcium signalling of radial glia characteristics in field 1 and field 2 at the different ages.

Age	Characteristics	Units	Field 1			Field 2		
			Mean	Std[1]	SEM[2]	Mean	Std[1]	SEM[2]
3 dpf	Number of events	#/4 min	7.44	4.59	0.36	5.50	3.51	0.21
	Amplitude	ΔF/F0	1.34	0.75	0.06	1.77	0.66	0.04
	Duration	(s)	6.94	5.58	0.47	10.46	6.72	0.46
	Rise time	(s)	2.64	2.28	0.19	3.05	2.15	0.15
	Synchrony	#/4 min	3.02	1.42	0.11	6.23	3.60	0.23
5 dpf	Number of events	#/4min	6.15	3.51	0.21	6.79	3.67	0.13
	Amplitude	ΔF/F0	1.65	0.69	0.04	1.83	0.61	0.02
	Duration	(s)	11.38	7.02	0.43	11.25	6.70	0.24
	Rise time	(s)	4.11	3.02	0.18	3.38	2.17	0.08
	Synchrony	#/4 min	10.30	4.35	0.23	12.98	4.82	0.25
7 dpf	Number of events	#/4min	8.43	4.91	0.26	7.29	4.82	0.21
	Amplitude	ΔF/F0	1.48	0.57	0.03	1.73	0.50	0.02
	Duration	(s)	9.27	6.01	0.35	9.96	5.44	0.24
	Rise time	(s)	3.40	2.59	0.15	3.38	2.28	0.11
	Synchrony	#/4 min	10.95	4.09	0.20	10.69	5.53	0.28

Std[1] Standard deviation

SEM[2] Standard error of the mean.

Table S3. Calcium signalling of radial glia characteristics of heptanol blocking test.

Characteristics	Units	Control			Heptanol		
		Mean	Std[1]	SEM[2]	Mean	Std[1]	SEM[2]
Number of events	#/4 min	35.86	20.29	0.18	4.77	3.43	0.11
Amplitude	ΔF/F0	2.34	1.05	0.01	1.57	0.79	0.02
Duration	(s)	13.32	8.77	0.08	13.09	11.75	0.37
Rise time	(s)	4.03	2.98	0.02	4.95	3.38	0.12
Synchrony	#/4 min	21.96	11.44	0.10	3.62	2.00	0.06

Std[1] Standard deviation

SEM[2] Standard error of the mean.

Table S4. Comparison of radial glia calcium characteristics in field 1 and 2 at the different ages.

Characteristics	Field	Kruskal Wallis test	SS	DF	MS	Chi-sq	P Value
Number of events	1	3, 5, 7 dpf	$1.6X10^{6}$	2	$7.99X10^{5}$	31.80	$1.25X10^{-7}$
	2	3, 5, 7 dpf	$4.9X10^{6}$	2	$2.44X10^{6}$	24.64	$4.46X10^{-6}$
	1vs2	f1-3-5-7 vs f2-3-5-7	$2.9X10^{7}$	5	$5.68X10^{6}$	63.36	$2.46X10^{-12}$
Amplitude	1	3, 5, 7 dpf	$1.1X10^{6}$	2	$5.62X10^{5}$	22.95	$1.04X10^{-5}$
	2	3, 5, 7 dpf	$9.16X10^{6}$	2	$4.58X10^{5}$	4.71	0.095
	1vs2	f1-3-5-7 vs f2-3-5-7	$5.48X10^{7}$	5	$1.10X10^{7}$	125.06	$1.04X10^{-25}$
Duration	1	3, 5, 7 dpf	$1.95X10^{6}$	2	$9.73X10^{5}$	45.99	$1.03X10^{-10}$
	2	3, 5, 7 dpf	$1.41X10^{6}$	2	$7.06X10^{5}$	7.59	0.0225
	1vs2	f1-3-5-7 vs f2-3-5-7	$3.01X10^{7}$	5	$6.01X10^{6}$	74.10	$1.44X10^{-14}$
Rise time	1	3, 5, 7 dpf	$1.06X10^{6}$	2	$5.28X10^{5}$	26.38	$1.87X10^{-6}$
	2	3, 5, 7 dpf	$6.98X10^{5}$	2	$3.49X10^{5}$	4.29	0.12
	1vs2	f1-3-5-7 vs f2-3-5-7	$1.22X10^{7}$	5	$2.44X10^{6}$	33.54	$2.94X10^{-6}$
Synchrony	1	3, 5, 7 dpf	$2.60X10^{7}$	2	$1.30X10^{7}$	360.66	$4.82X10^{-79}$
	2	3, 5, 7 dpf	$1.95X10^{7}$	2	$9.74X10^{6}$	227.86	$3.31X10^{-50}$
	1vs2	f1-3-5-7 vs f2-3-5-7	$1.84X10^{8}$	5	$3.69X10^{7}$	586.15	$1.99X10^{-124}$

Characteristics	Field	Multicompare		Group 1	Group 2	Mean diff	P value	Summary
Number of events	1	3 dpf	5 dpf	7.88	59.67	111.46	0.0190	*
		3 dpf	7 dpf	-92.76	-42.46	7.83	0.1174	ns
		5 dpf	7 dpf	-144.61	-102.13	-59.65	0.0000	***
	2	3 dpf	5 dpf	-235.18	-155.91	-76.64	0.0000	***
		3 dpf	7 dpf	-248.85	-165.54	-82.23	0.0000	***
		5 dpf	7 dpf	-68.06	-9.63	48.80	0.9210	ns
	1 vs 2	3 dpf	3 dpf	98.76	295.86	492.93	0.0003	***
		5 dpf	5 dpf	-236.78	-104.48	27.83	0.2149	ns
		7 dpf	7 dpf	43.91	176.92	309.93	0.0021	*
Amplitude	1	3 dpf	5 dpf	-156.42	-104.92	-53.43	0.0000	***
		3 dpf	7 dpf	-113.79	-62.80	-11.81	0.0109	*
		5 dpf	7 dpf	0.18	42.12	84.07	0.0488	*
	2	3 dpf	5 dpf	-119.84	-42.40	35.04	0.4047	ns
		3 dpf	7 dpf	-73.03	9.11	91.24	0.9635	ns
		5 dpf	7 dpf	-7.28	51.51	110.29	0.0998	ns
	1 vs 2	3 dpf	3 dpf	-623.89	-427.44	-230.99	0.0000	***
		5 dpf	5 dpf	-339.74	-211.30	-82.86	0.0000	***
		7 dpf	7 dpf	-425.12	-289.62	-154.51	0.0000	***
Duration	1	3 dpf	5 dpf	-193.47	-143.65	-93.83	0.0000	***
		3 dpf	7 dpf	-134.29	-85.26	-36.22	0.0001	***
		5 dpf	7 dpf	17.94	58.40	98.86	0.0021	*
	2	3 dpf	5 dpf	-137.59	-59.21	19.16	0.1795	ns
		3 dpf	7 dpf	-79.79	3.31	86.41	0.9952	ns
		5 dpf	7 dpf	4.56	62.52	120.49	0.0308	*
	1 vs 2	3 dpf	3 dpf	-561.81	-365.22	-168.63	0.0000	***
		5 dpf	5 dpf	-131.80	-3.97	123.87	1.0000	ns
		7 dpf	7 dpf	-235.58	-102.55	30.47	0.2391	ns
Rise time	1	3 dpf	5 dpf	-155.96	-106.84	-57.72	0.0000	***
		3 dpf	7 dpf	-110.18	-61.41	-12.64	0.0089	*
		5 dpf	7 dpf	5.65	45.43	85.21	0.0203	*

	2	3 dpf	5 dpf	-142.50	-66.58	9.35	0.0994	ns
		3 dpf	7 dpf	-138.42	-58.22	21.98	0.2046	ns
		5 dpf	7 dpf	-47.50	8.36	64.22	0.9345	ns
	1 vs 2	3 dpf	3 dpf	-334.33	-143.06	48.21	0.2710	ns
		5 dpf	5 dpf	-20.19	101.97	224.13	0.1637	ns
		7 dpf	7 dpf	-156.87	-27.75	101.37	0.9902	ns
Synchrony	1	3 dpf	5 dpf	-469.57	-411.08	-352.60	0.0000	***
		3 dpf	7 dpf	-505.31	-447.70	-390.08	0.0000	***
		5 dpf	7 dpf	-82.27	-36.61	9.05	0.1447	ns
	2	3 dpf	5 dpf	-415.54	-359.55	-303.57	0.0000	***
		3 dpf	7 dpf	-292.66	-237.13	-181.61	0.0000	***
		5 dpf	7 dpf	72.81	122.42	172.02	0.0000	***
	1 vs 2	3 dpf	3 dpf	-0.51	-0.35	-0.20	0.0000	***
		5 dpf	5 dpf	-0.39	-0.27	-0.15	0.0000	***
		7 dpf	7 dpf	-0.06	0.05	0.17	0.0008	***

p-value <0.05*, 0.01**, 0.001***

Table S5. Comparison of radial glia blockage by heptanol.

Characteristics	Mann-Whitney test	Mean	SD	Z	P Value	Summary
Number of events	control vs heptanol	$6.5X10^{+6}$	$1.2X10^{+5}$	49.33	0	****
Amplitude	control vs heptanol	$7.1X10^{+6}$	$1.3X10^{+5}$	23.39	0	****
Duration	control vs heptanol	$6.5X10^{+6}$	$1.2X10^{+5}$	5.89	$1.9X10^{-9}$	***
Rise time	control vs heptanol	$5.7X10^{+6}$	$1.1X10^{+5}$	6.35	$1.0X10^{-10}$	***
Same time	control vs heptanol	$6.95X10^{+6}$	$1.2X10^{+5}$	53.49	0	****

p-value α 0.05*, 0.01**, 0.001*** 0****, SD = standard deviation., Z = z-value.

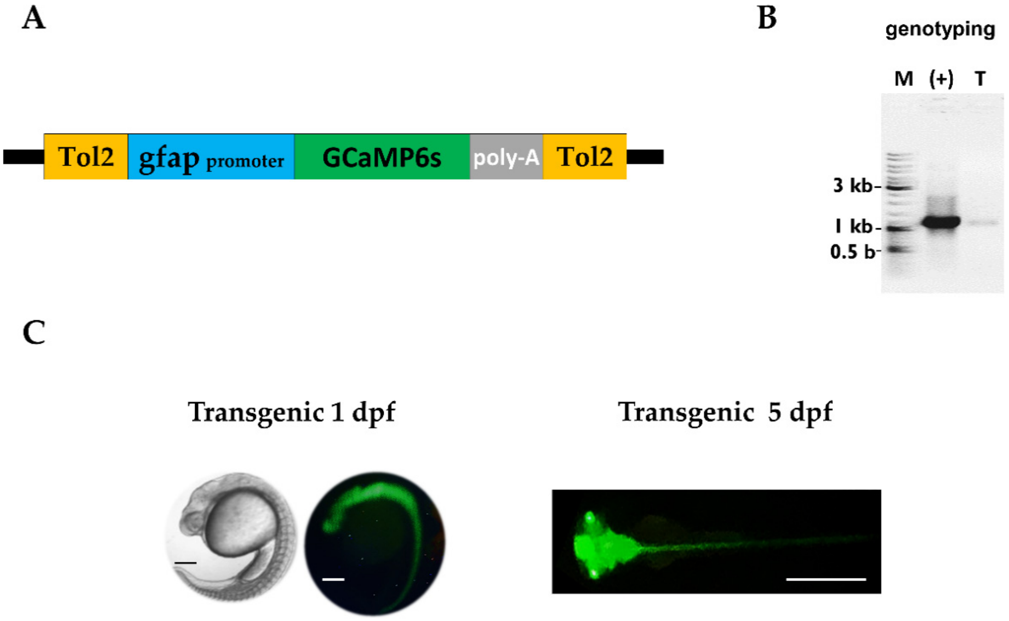

Figure S1. Tg(pTol2-gfap-GCaMP6s). A. Scheme of the pTol2-gfap-GCaMP6s cassette. B. Genotyping by PCR from tail of adult fish. C. Transgenic larvae imaged by fluorescence microscopy at 1 dpf and 5 dpf (lateral and dorsal view respectively). Kb, kilobases; M(ladder); (+), positive control (amplicon from GCaMP6s plasmid); T, amplicon from transgenic adult zebrafish tail; dpf, days postfertilization. Scale bar 500 µm and 1 mm.

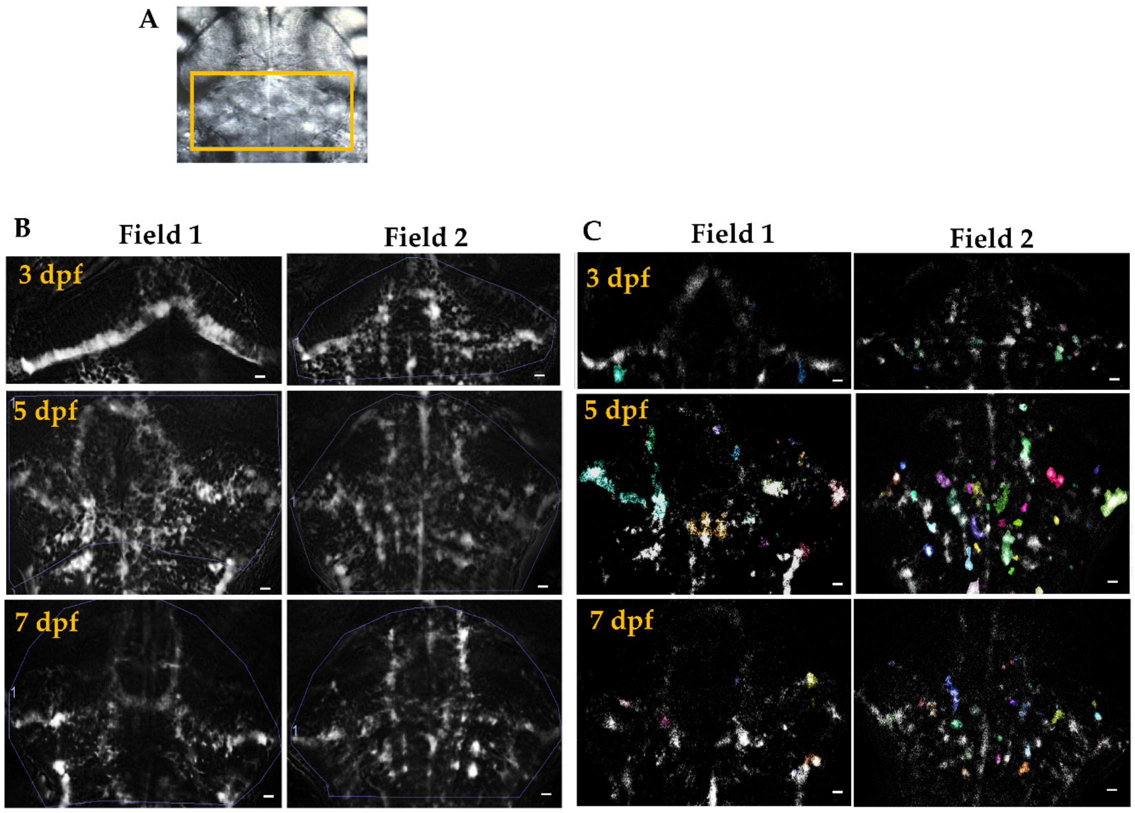

Figure S2. Anatomic zone of video recordings. A. Bright field image of the larva in dorsal view. (Yellow line square cerebellum) B. Cerebellum z-projection of the ventral and dorsal zones classified as field 1 and field 2 for video recordings. C. Radial glia cells with calcium activity (multicoloured) in a sample time point of record.
